# Supplementary material for: SlTDC1 Overexpression Promoted Photosynthesis in Tomato under Chilling Stress by Improving CO2 Assimilation and Alleviating Photoinhibition
Source: Int J Mol Sci. 2023 Jul 3;24(13):11042. doi: 10.3390/ijms241311042 (PMC10341946; doi:10.3390/ijms241311042)
Supplement: Supplementary file 1 [file ijms-24-11042-s001.zip › ijms-2456144-supplementary.pdf]

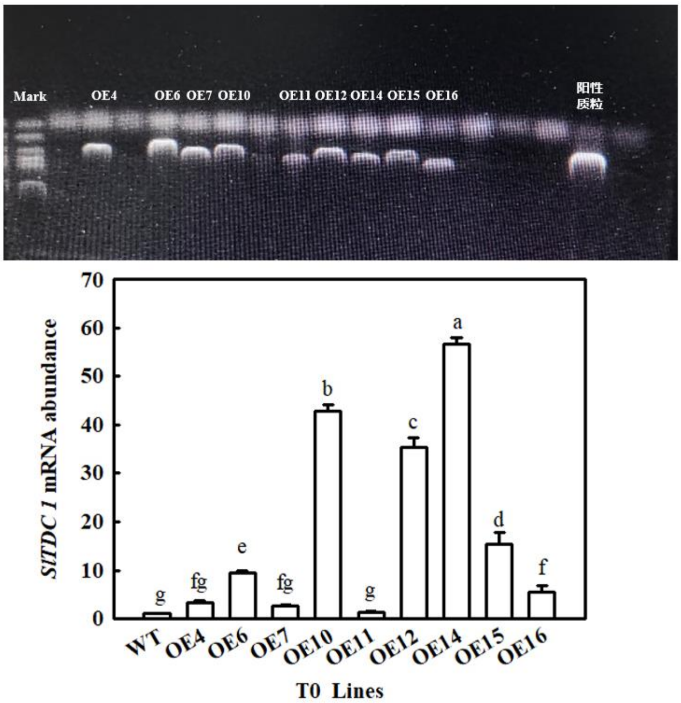

Figure S1 Determination of gene expression in transgenic lines. All values shown are mean ± SD (n = 3). a-g indicate that mean values are significantly different among samples ($p < 0.05$).
